# Completion of Advance Directives and Documented Care Preferences During the Coronavirus Disease 2019 (COVID-19) Pandemic

**DOI:** 10.1001/jamanetworkopen.2020.15762

**Published:** 2020-07-20

**Authors:** Catherine L. Auriemma, Scott D. Halpern, Jeremy M. Asch, Matthew Van Der Tuyn, David A. Asch

**Affiliations:** 1 Palliative and Advanced Illness Research (PAIR) Center, University of Pennsylvania, Philadelphia; 2 Department of Medicine, University of Pennsylvania, Philadelphia; 3 Leonard Davis Institute of Health Economics, University of Pennsylvania, Philadelphia; 4 Department of Medical Ethics and Health Policy, University of Pennsylvania, Philadelphia; 5 Center for Health Care Innovation, University of Pennsylvania, Philadelphia; 6 Corporal Michael J. Crescenz VA Medical Center, Philadelphia, Pennsylvania

## Abstract

This cohort study examines changes in completion of and expressed preferences on an online advanced directive platform during the coronavirus 2019 (COVID-19) pandemic.

## Introduction

Fewer than one-third of US residents have completed an advance directive (AD) to guide care when seriously ill.^1^ Clinician-focused efforts to increase AD completion, such as Medicare payments for advance care planning (ACP), have been ineffective.^2^ In contrast, patient-facing interventions that enable independent completion of ADs show early promise.^3^ Self-service platforms also reveal changes in demand for ADs and preferences for future care. We sought to quantify changes in patient completion of ADs and expressed preferences during the coronavirus disease 2019 (COVID-19) pandemic by monitoring users of a web-based AD platform from January 2019 to April 2020.

## Methods

We conducted a prospective cohort study of users of OurCareWishes.org, a free tool designed to guide patients and families through ACP. We evaluated monthly rates of AD completion, number of goal-setting modules completed, and distributions of preferences for care. We designated the pre–COVID-19 period as January 1, 2019, to January 31, 2020 (ie, the date the United States declared a public health emergency), and the COVID-19 period from February 1 to April 30, 2020. There were no special dissemination efforts of the OurCareWishes.org platform during the COVID-19 period. This study was approved by the University of Pennsylvania institutional review board. Informed consent was waived for this minimal risk study. This report follows Strengthening the Reporting of Observational Studies in Epidemiology (STROBE) reporting guidelines for cohort studies.

We used *t* tests, χ^2^ tests, and Kruskal-Wallis tests to compare patient age, rates of module completion, and ordered goals of care, respectively, before and during COVID-19. We used multivariable logistic regression to calculate estimated probabilities and odds of completing modules in each period, rating certain priorities near end of life as extremely important, and perceiving certain health states as similar to or worse than dying comfortably. All models were adjusted for age. Models assessing AD choices also included self-reported health (ie, good health, some health problems, at least 1 serious health condition, and terminal illness). Analyses were conducted using Stata version 15.1 (StataCorp). Statistical significance was set at *P* < .05, and all tests were 2-tailed.

## Results

During the pre–COVID-19 period, there were a total of 424 users, with a median (interquartile range) of 26 (22-30) monthly new users, 5 (3-6) monthly returning users, and 31 (28-34) monthly total users. During the COVID-19 period, corresponding medians (IQRs) were 133 (71-207), 21 (11-39), and 154 (82-246), respectively. These figures represent a 4.9-fold increase in monthly users in the COVID-19 period compared with the pre–COVID-19 period, with considerable increases from February to April (Figure). Total users in February, March, and April 2020 were 82, 154, and 246, respectively, for a total of 482 users. Individuals using the service in the COVID-19 period were slightly younger than those using it in the pre–COVID-19 period (mean age, 49.3 [95% CI, 47.8-50.9] years vs 51.8 [95% CI, 50.2-53.5] years; *P* = .03), with better self-reported health (Kruskal-Wallis *H*, 4.06; *P* = .04). Of 9 optional modules, completion rates increased during COVID-19 for 5 modules, ie, identifying goals of care (110 [25.9%] vs 201 [41.7%]; *P* < .001), important end-of-life priorities (101 [23.8%] vs 181 [37.6%]; *P* < .001), health state ratings (106 [25.0%] vs 198 [41.1%]; *P* < .001), organ donation (110 [25.9%] vs 186 [38.6%]; *P* < .001), and wishes for one’s final days (54 [12.7%] vs 109 [22.6%]; *P* < .001).

**Figure.**
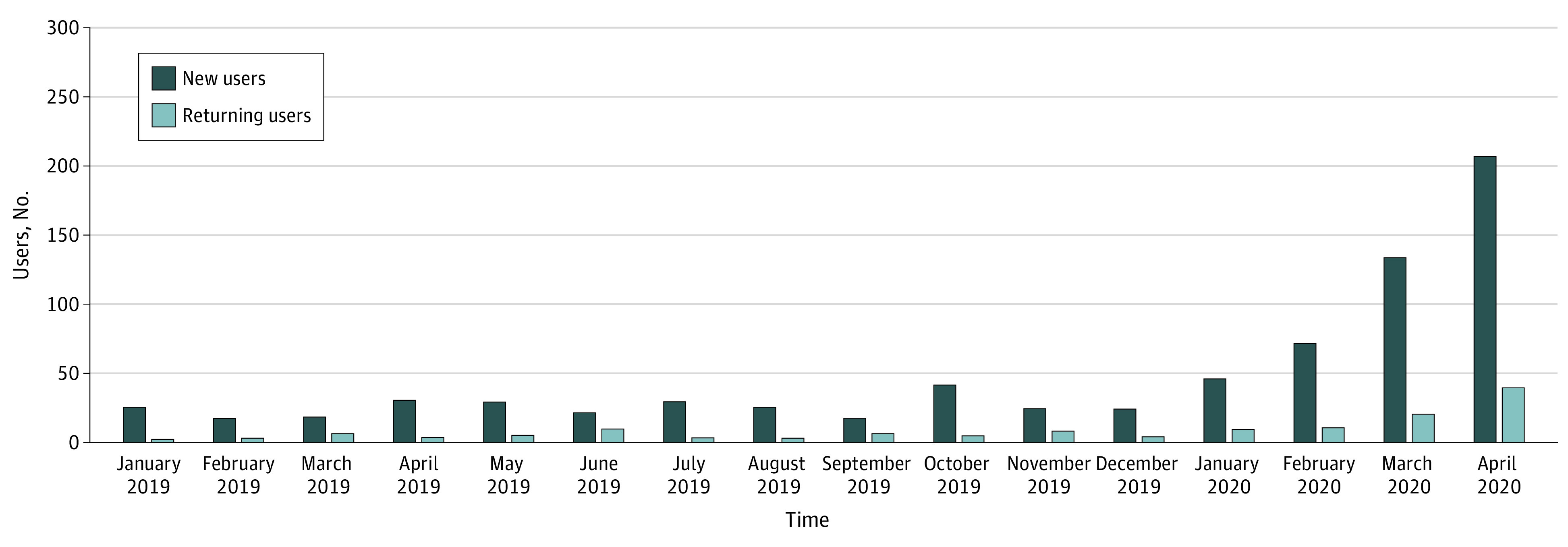
Advance Directive Completion by Month Among New and Returning Users of the Online Platform

Patients’ goals of care and ratings of end-of-life priorities were similar between periods (Table). The odds of rating 4 of 10 health states (ie, constant confusion, requiring a feeding tube, incontinence, and needing full-time care) as similar to or worse than dying comfortably increased in the COVID-19 period compared with the pre–COVID-19 period (constant confusion: 93.4% [95% CI, 89.9%-97.0%] vs 81.9% (95% CI, 74.4%-89.4%]; *P* = .03; requiring a feeding tube: 88.7% [95% CI, 84.2%-93.1%] vs 75.6% [95% CI, 67.0%-84.2%]; *P* = .01; incontinence: 87.3% [95% CI, 82.6%-92.0%] vs 73.7% [64.9%-82.6%]); *P* = .01; needing full time care: 93.9% [95% CI, 90.6%-97.2%] vs 83.6% [76.2%-91.0%]; *P* = .01) (Table), whereas corresponding odds did not decrease for any health states.

**Table.** Patient Responses to Advance Directive Modules on Goals of Care, Priorities Near the End of Life, and Ratings of Health States

Choice	Estimated probability, % (95% CI)^a^		*P* value
	Pre–COVID-19 period	COVID-19 period	
Overall goals of care if sick and not getting better (n = 293)^b^			
Comfort and QOL	20.5 (14.1-26.9)	24.5 (18.9-30.1)	.43
Comfort and QOL with treatments that may prolong life without hospitalization	37.3 (31.4-43.6)	38.7 (33.1-44.3)	
Comfort and QOL with hospitalization but no life support treatments	14.4 (10.2-18.7)	13.4 (9.5-17.3)	
Comfort and QOL with a short period of life support in a hospital	21.0 (14.9-27.2)	18.0 (13.3-22.6)	
Prolonged life	6.7 (3.3-10.2)	5.4 (2.8-8.0)	
Important priorities near the end of life (n = 272)^c^			
Having loved ones around me	82.2 (74.9-89.4)	74.7 (68.5-80.9)	.14
Being alert enough to talk with loved ones	71.6 (62.7-80.5)	76.8 (70.6-83.1)	.34
Not spending a lot of time on life support machines	65.2 (55.8-74.5)	74.2 (68.1-80.4)	.11
Not spending a lot of time in an ICU	42.2 (32.6-51.7)	46.6 (39.3-53.8)	.48
Not having pain	41.3 (31.7-51.0)	39.6 (32.5-46.7)	.78
Having someone from my religion or spiritual faith visit me	27.8 (18.7-36.4)	20.3 (14.3-26.2)	.17
Living as long as possible	25.3 (17.1-33.6)	19.2 (13.5-24.8)	.22
Dying at home	20.4 (12.7-28.2)	23.3 (17.0-29.5)	.58
Health states perceived as similar to or worse than dying comfortably (n = 283)^d^			
I cannot live outside a medical facility	91.1 (85.3-96.9)	92.2 (88.4-95.9)	.77
I rely on a breathing machine to live	89.0 (82.7-95.4)	94.3 (91.0-97.5)	.12
I need care all the time	83.6 (76.2-91.0)	93.9 (90.6-97.2)	.01
I am confused all the time	81.9 (74.4-89.4)	93.4 (89.9-97.0)	.03
I cannot get out of bed	79.9 (71.8-88.0)	83.8 (78.6-88.9)	.42
I rely on a feeding tube to live	75.6 (67.0-84.2)	88.7 (84.2-93.1)	.01
I cannot control by bladder or bowels	73.7 (64.9-82.6)	87.3 (82.6-92.0)	.01
I have to stay home all day	67.4 (57.9-77.0)	74.4 (68.1-80.6)	.23
I am in moderate pain all the time	65.9 (56.4-75.3)	68.1 (61.6-74.7)	.70
I am wheelchair bound	57.5 (47.8-67.1)	58.3 (51.5-65.0)	.89

Abbreviations: COVID-19, coronavirus disease 2019; ICU, intensive care unit; QOL, quality of life.

^a^ Estimated using multivariable logistic regression adjusting for patient age and patient-reported health status.

^b^ Probability of electing stated preference for care. Comparison of response distribution in pre–COVID-19 period with COVID-19 period performed using Kruskal-Wallis test.

^c^ Probability of rating end-of-life goal as extremely important.

^d^ Probability of rating health state as equal to or worse than dying comfortably.

## Discussion

This study reveals a 4.9-fold increase in online AD completion as well as more comprehensive completion since the onset of the COVID-19 pandemic in the absence of contemporaneous efforts to increase uptake of the ACP platform. Distributions of preferences were largely unchanged. Study limitations include unmeasured trends that may affect AD demand. Furthermore, while the platform is publicly available, it is largely used by patients within a single health system. The increased demand for AD documentation might be explained by an increased sense of AD importance owing to COVID-19–induced hospital visitation restrictions,^4^ calls for clinicians to promote ACP,^5^ or because COVID-19 has provided new motivation for patients who have long wanted to complete ADs but previously failed to do so.^6^

## References

[1] Yadav KN, Gabler NB, Cooney E, Approximately one in three US adults completes any type of advance directive for end-of-life care. Health Aff (Millwood). 2017;36(7):1244-1251. doi:10.1377/hlthaff.2017.0175 28679811

[2] Ashana DC, Halpern SD, Umscheid CA, Kerlin MP, Harhay MO Use of advance care planning billing codes in a retrospective cohort of privately insured patients. J Gen Intern Med. 2019;34(11):2307-2309. doi:10.1007/s11606-019-05132-1 31367871 PMC6848717

[3] Sudore RL, Schillinger D, Katen MT, Engaging diverse English- and Spanish-speaking older adults in advance care planning: the PREPARE randomized clinical trial. JAMA Intern Med. 2018;178(12):1616-1625. doi:10.1001/jamainternmed.2018.4657 30383086 PMC6342283

[4] Block BL, Smith AK, Sudore RL During COVID-19, outpatient advance care planning is imperative: we need all hands on deck. J Am Geriatr Soc. Published May 2, 2020. doi:10.1111/jgs.16532 32359075 PMC7267338

[5] Curtis JR, Kross EK, Stapleton RD The importance of addressing advance care planning and decisions about do-not-resuscitate orders during novel coronavirus 2019 (COVID-19). JAMA. 2020;323(18):1771-1772. doi:10.1001/jama.2020.4894 32219360

[6] Halpern SD Shaping end-of-life care: behavioral economics and advance directives. Semin Respir Crit Care Med. 2012;33(4):393-400. doi:10.1055/s-0032-1322403 22875386

